# Habitat-Specific Morphological Variation among Threespine Sticklebacks (*Gasterosteus aculeatus*) within a Drainage Basin

**DOI:** 10.1371/journal.pone.0021060

**Published:** 2011-06-15

**Authors:** Mike M. Webster, Nicola Atton, Paul J. B. Hart, Ashley J. W. Ward

**Affiliations:** 1 School of Biology, University of Leicester, Leicester, United Kingdom; 2 School of Biology, University of St Andrews, St Andrews, United Kingdom; 3 School of Biological Sciences, University of Sydney, Sydney, Australia; Texas A&M University, United States of America

## Abstract

Habitat-specific morphological variation, often corresponding to resource specialization, is well documented in freshwater fishes. In this study we used landmark based morphometric analyses to investigate morphological variation among threespine sticklebacks (*Gasterosteus aculeatus* L.) from four interconnected habitat types within a single lowland drainage basin in eastern England. These included the upper and lower reaches of the river, the estuary, a connected ditch network and a coastal salt marsh. We found significant habitat-specific differences in morphology, with three axes of variation describing differences in orbit diameter, body depth, caudal peduncle shape and pectoral fin positioning as well as variation in relative dorsal and pelvic spine size. Interestingly, the ditch system, an artificial and heavily managed habitat, is populated by sticklebacks with a characteristic morphology, suggesting that human management of habitats can in some circumstances lead to morphological variation among the animals that inhabit them. We discuss the mechanisms that conceivably underlie the observed morphological variation and the further work necessary to identify them. Finally, we consider the implications of habitat-specific body shape variation for the behavioural ecology of this ecologically generalist species.

## Introduction

Species whose range encompasses multiple habitats often exhibit functional trade-offs corresponding to local specialization in resource use. For many of these species, morphology has been shown to correlate strongly with habitat specialization, with even subtle variation in body shape seen to be correlated with fitness parameters such as foraging success, growth rate and body condition. Selection pressures operating within different habitats or habitat units favour specific traits. This can lead to fitness trade-offs, since traits that are well suited to the conditions found in one location may be ill-suited to those found in others. While such traits can benefit their bearer by increasing the efficiency with which specific resources can be exploited, giving them a competitive advantage that ultimately translates into greater fitness, the cost of acquiring such traits is reduced efficiency in the use of other available resources [1], [2], [3], [4].

Morphological variation corresponding to differences in habitat and resource use have been described in a diverse array of animal groups, including insects [5], gastropods [6]; amphibians [7]; reptiles [8], [9]; mammals [10] and birds [11], [12]. In fishes, intraspecific morphological variation has been described within populations of numerous species occurring across environmental gradients such as flow regime, water depth, water chemistry, substrate type, predation risk and prey assemblage, in a diverse range of gross habitat types, including high latitude post glacial lakes [13], [14], [15], [16], [17], [18], [19], [20], [21], [22], [23], [24], [25], reviewed by [26], [27], drainage networks [28], [29], [30], , surface-subterranean systems [33] and coastal environments [31].

As a first step towards identifying putative habitat specialization within environmentally heterogeneous drainage basin systems this study aimed to quantify morphological variation among threespine sticklebacks (*Gasterosteus aculeatus* L.) collected from discreet but connected habitat units comprising a lowland drainage basin. These included the upper and lower reaches of the river, the estuary, a man-made drainage ditch network and a coastal salt marsh. These habitats varied markedly both in their physical characteristics, such as channel size, flow rate, turbidity and structural complexity, and also in the assemblages of predators that they contained.

The threespine stickleback was selected as the focus of our study because it is an ecological generalist, occurring across a diverse range of freshwater, estuarine and coastal habitats throughout the temperate Northern hemisphere, and because it is known to exhibit substantial variation in resource use and morphology, both within and between populations [34]. This variation ranges in magnitude from the much studied benthic and limnetic species pair [18] to subtle intra-population variation in prey resource use [35]. This paper outlines a study designed to test the prediction that sticklebacks collected from markedly different but connected habitat components of our focal drainage basin would exhibit significant morphological variation. Specifically, we predicted that we would see variation in both body depth and spine size between fish from habitats that differed in fish predator assemblage. Local responses to differences in the intensity and nature of predation risk is a significant driver of morphological variation in many species, and has been recorded in a range of taxa [5], [6], [15], [36], [37], [38], [39], [40].

In sticklebacks, large relative spine size is an adaptation to mitigate predation by gape limited predators, and has been shown to vary between population in this species, as a function of predation risk [39], [40]. Body depth varies as a trade-off between predation risk and swimming energetics, where deeper bodies reduce susceptibility to predation from gape limited predators, while shallower bodies leader to greater swimming efficiency through reduced drag. Many species possess the capacity for phenotypic plasticity in body depth as a function of local predation pressure [15], [36]. In our study we predicted that fish from the river and estuary habitats, which contain relatively high densities of piscivorous fish predators, such as Eurasian perch (*Perca fluviatilis*) and northern pike (*Esox lucius*) would have relatively larger spines and deeper bodies compared to the fish from the ditches and salt marsh, which contain no fish predators.

A second aim of our study was to gain insight into the morphological responses of fish living in altered habitats. Our study drainage contained not only natural elements, the river, estuary and salt marsh, but also an entirely man-made and intensively managed habitat, an extensive drainage ditch network constructed in its current form in the mid 17^th^ century [41] and maintained through regular dredging. Given the marked physical and ecological differences between the ditch network and the natural river channel (see Table 1) to which it is connected at multiple points, we further predicted that we would see morphological variation between fish from these habitats.

**Table 1:** Overview of environmental parameters at the six collection sites.

	Estuary	River	River	Ditch 1	Ditch 2	Salt marsh
		(lower)	(upper)			
**Coordinates**	53°25′02N	53°22′11N	53°19′016N	53°25′05N	53°26′06N	53°23′58N
	0°12′17E	0°11′22E	0°08′16E	0°11′54E	0°10′37E	0°10′52E
**Maximum channel width (m)**	20^a^	10	6	2	2	300 m×30
**Maximum channel depth (m)**	6^a^	3	1.5	0.6	0.3	1.2
**Maximum flow rate (m/s)**	0.1^a^	0.3	0.5	0.05	0.01	none
**Turbidity** b	0.3	3	3	0.5	0.5	1
**Salinity**	1.000–	1.000	1.000	1.006–	1.006–	1.022–
**(specific gravity)**	1.024^a^			1.014^c^	1.020^c^	1.035^c^
**Predator assemblage**						
**Fishes** d	Present^e^	Present	Present	Absent	Absent	Absent
**Birds** f	Present	Present	Present	Present	Present	Present
**Insect larvae** g	Absent	Present	Present	Absent	Absent	Absent

a Determined by tidal cycle.

b Maximum visual range, based on estimated Secchi depth.

c Observed range over 12 month period (2005–2006), determined by variation in rates of precipitation and evaporation.

d Including brown trout, *Salmo trutta*; Eurasian perch, *Perca fluviatilis*, northern pike, *Esox Lucius*.

e Eurasian perch and northern pike present at low tide only.

f Including grey heron, *Ardea cinerea*; kingfisher, *Alcedo atthis* and little egret, *Egretta garzetta*.

g Including emperor dragonfly, *Anax imperator* and great diving beetle *Dytiscus marginalis*.

## Methods

### Collection and housing

Sticklebacks were collected using dipnets from six sites in the Great Eau drainage in Lincolnshire, eastern England during September 2005. This drainage consists of four distinct but interconnected habitat types; the river channel, the estuary, a network of man-made drainage ditches, and a coastal salt marsh system, which differ markedly in a number of environmental parameters (Table 1). Sticklebacks are common in all of the four habitat types. The six sites were (1) a 250 m long stretch of the estuary (n = 21 fish), (2) a non-tidal 50 m stretch of the river 7.5 km upstream from the estuary, (‘the lower river site’, n = 37), (3) a 50 m stretch of river 7.8 km further upstream (15.3 km upstream from the estuary, ‘the upper river site’, n = 41), (4) a 50 m stretch of ditch approximately 200 m from its confluence with the estuary (‘ditch site 1’, n = 28), (5) a 50 m stretch of ditch in the same network, 2.5 km away from, and connected to ditch site 1 (‘ditch site 2’, n = 33) and (6) a 9000 m^2^ pool in the salt marsh system that was intermittently connected to the estuary by a tidal creek system (n = 56). Coordinates for the sites of collection are presented in Table 1. All fish were fully plated. There were no size differences between fish from the six sites (mean +/− standard error = 37.9 mm+/−0.32 mm, range = 36 to 40 mm, one-way ANOVA: F_(5, 210)_ = 0.60, P = 0.72).

The fish were transported to the laboratory, where they were held in 45 L aquaria at 12°C in groups of approximately 20. Fish from different habitats were held separately. They were fed daily with frozen bloodworm (Chironomus sp. larvae). Fish were photographed for morphological analyses seven days after capture and were food deprived for 24 hours before being photographed. They were photographed while alive, and afterwards were retained in the lab for use in a separate behavioral experiment [42]. Photographing took around 30 s per fish, after which they were returned to different holding tanks. None were observed to suffer injury or exhibit unusual behavior after being photographed.

### Morphological analyses

**Figure 1:**
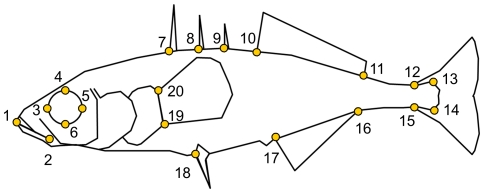
Location of the landmarks used in the morphological analysis. Line diagram of the left flank of a threespine stickleback, showing the locations of the 20 landmarks that were used in the morphological analyses. These landmarks correspond to: (1) the tip of the premaxilla, (2) the axis of the jaws, (3–6) the anterior-most, uppermost, posterior-most and lowermost point of the orbital circumference, (7–9) the posterior most edge of the first, second and third dorsal spines, at the points where they emerge from the dorsal surface, (10–11) the anterior- and posterior-most edges of the dorsal fin, at the points where it emerges from the dorsal surface, (12) the beginning of the caudal fin, where the membrane contacts the dorsal surface, (13–14) the upper- and lowermost points of the hypural fan, (15) the beginning of the caudal fin, where the membrane contacts the ventral surface, (16–17) the posterior- and anterior-most edges of the anal fin, at the points where it emerges from the ventral surface, (19) the posterior most edge of the left pelvic spine, (19–20) the lower and uppermost points of the pectoral fin base.

Each fish was placed on its right flank within a groove in a polystyrene block, to prevent deformation of the body along its length and a digital image of the left flank was captured. The camera tripod and polystyrene block weresecured to a bench in order to ensure that the camera position was consistent across samples. Morphology was analysed using landmark-based geometric morphometrics and thin-plate spline analysis. The program TpsDIG [43] was used to capture the x and y coordinates of 20 landmarks from each image (Figure 1). The landmarking procedure was performed blind, so that the researcher who digitised the landmark locations was not aware of the site of collection of the fish. The data generated by TpsDIG was then transferred to the program TpsRelw [43], which uses principle component analysis (PCA) to describe shape variation in the sample by comparing the landmark coordinates of each individual with the landmark coordinates of all of the other individuals in the sample. In order to control for allometry we used a MANCOVA to obtain residuals of body size (length to the nearest 0.1 mm) and the partial warp and uniform score values. This procedure was performed using SPSS. These size-residual principal components were then entered together as parameters into a multivariate discriminant function analysis (DFA), also using SPSS. The sites of capture of the fish were used as grouping variables. The DFA combined all the scores obtained from TpsRelw into discriminant functions, hereafter referred to as morphological indices, with a unique variable for each individual that maximally discriminates between the groups. TpsRegr [43] was used to visualize body shape variation. This program performs a regression between the coordinates captured by TpsDIG and the discriminant variables obtained from the DFA to produce grid deformation plots. To determine whether morphology varied between fish from the different habitat types a one-way ANOVA with Tukey post-hoc analyses was performed for each of the morphological indices produced by the DFA, with site of collection as the grouping variable.

### Controlling for landmark precision

**Figure 2:**
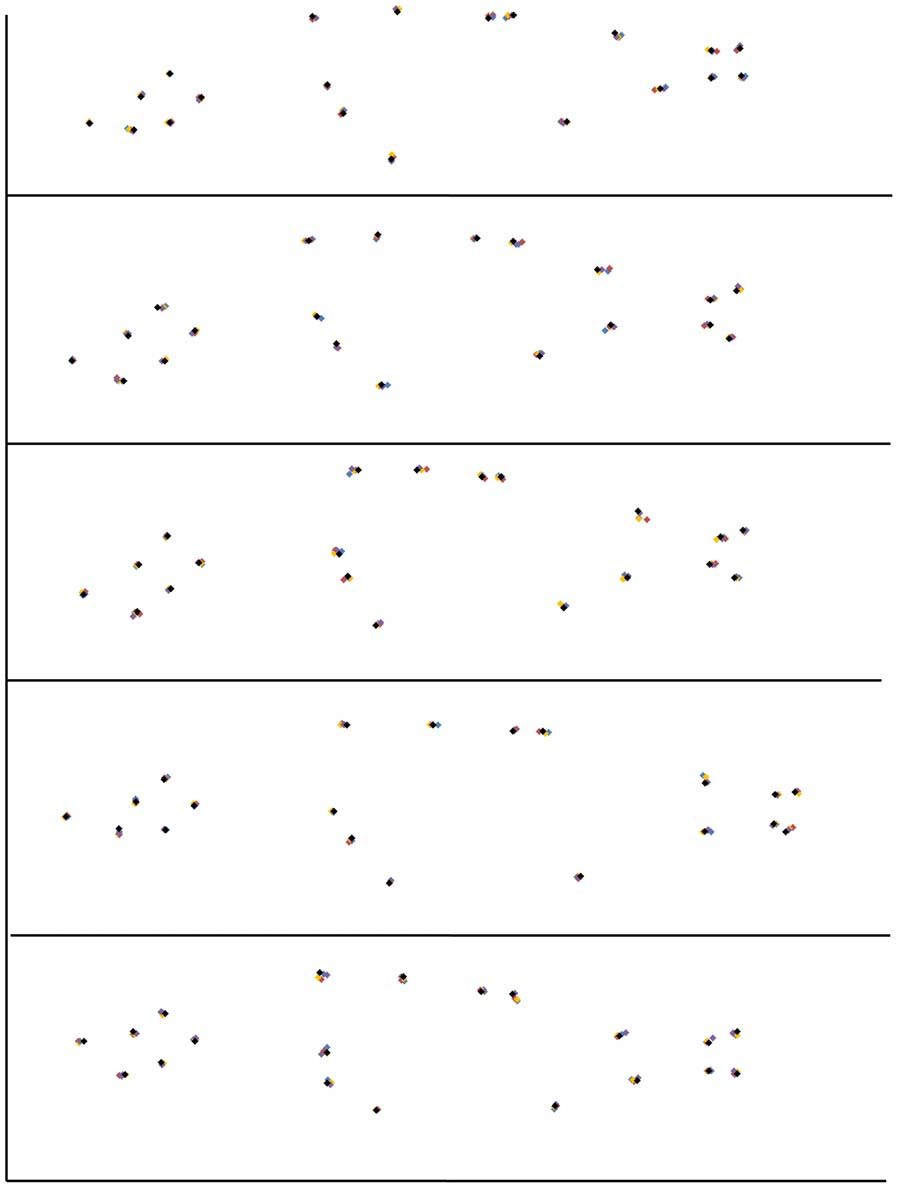
Plots showing consistency of landmark location. In order to confirm that landmarks could be identified precisely and consistently, both within and between samples, we repeatedly digitised landmarks on the same images. Five images were selected at random. For each image, the same 20 landmarks were digitised each day, for five consecutive days (see Methods and Figure 1 for discussion of the landmark selection criteria). The order in which each image was landmarked was randomised for each day. Landmark locations for days 1 to 5 are represented by blue, red, yellow, purple and black markers respectively. Each image represents a fish 36–40 mm in length.

In order to confirm that landmarks could be identified consistently, both within and between samples, we repeatedly digitised landmarks on the same images, in order to determine landmark precision. Five images were selected at random. For each image, the same 20 landmarks were digitised each day, for five consecutive days. The order in which the images were landmarked was randomised for each day. Figure 2 shows that landmarks could be readily identified with minimal error, ensuring that the morphological variation described below in the results section is due to between-habitat variation in body shape, and does not reflect sampling error.

### Dorsal and pelvic spine relative size

TpsDIG was also used to measure the length of the first dorsal spine, the left pelvic spine and the standard length of each fish. Proportional spine length was determined by dividing spine length by body length. Using body length-adjusted spine measurements alone is controversial, since the effectiveness of spines against gape-limited predators is strongly dependent upon body depth, the relative distance between the base of the pelvic spines and the dorsal surface (Bell & Foster, 1994). In analysing both spine length and whole body morphology together both subjects of variation were accounted for. For each spine measure, the proportional length values (normalised by arcsine transformation) were compared between sites of collection using General Linear Models (GLMs), with the whole body morphological indices included as covariates.

## Results

### Body shape

The DFA revealed five morphological indices describing 37.5, 26.6, 22.1, 9.4 and 4.4% of the morphological variation in the sample. We consider first three of these further, below.

#### Morphological index 1

**Figure 3:**
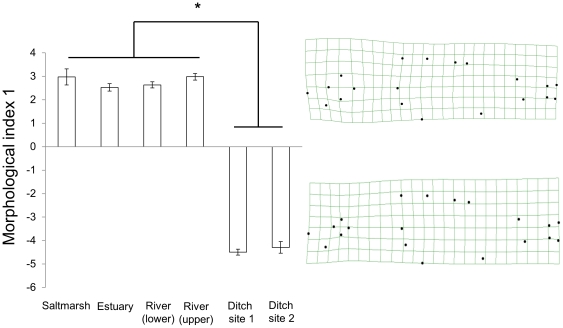
Morphological index 1. The mean scores (+/− standard error) of fish from each of the six collection sites for the first morphological index, describing described 37.7% of the morphological variation in the sample. Morphological index 1 was primarily associated with variation in orbit diameter. Orbit diameter increased with decreasing morphological index score. The deformation plots on the right of the figure show the morphologies associated with the positive-most and negative-most scores. Tukey post-hoc analyses: * indicates P<0.05.

The first morphological index described variation in orbit diameter, which varied significantly between sites (one-way ANOVA: F_(5, 215)_ = 384.44, P<0.001, Fig. 3). Individuals with negative indices had relatively larger eyes compared to those with positive indices. Smaller eyes were typical of fish from the two ditch sites, while fish from the estuary, river and salt marsh sites had larger eyes. We saw no variation between fish from the two ditch sites (Tukey post-hoc: P = 0.98), nor between the estuary, river and salt marsh sites (estuary vs. lower and upper river sites and salt marsh site: P = 0.55, 0.99 and 0.80 respectively; lower and upper river site versus salt marsh site: P = 0.67, 0.88 respectively, lower versus upper river site: P = 0.57). Fish from the salt marsh, the estuary and river sites differed significantly from fish from the two ditch sites (P<0.001 in all cases).

#### Morphological index 2

**Figure 4:**
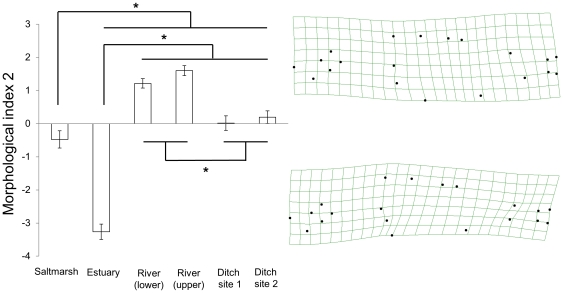
Morphological index 2. The mean scores (+/− standard error) of fish from each of the six collection sites for the second morphological index, describing described 26.6% of the morphological variation in the sample. Morphological index 2 includes variation in body depth and the robustness of the caudal peduncle and the size and angle of the base of the pectoral fin. The deformation plots on the right of the figure show the morphologies associated with the positive-most and negative-most scores. Tukey post-hoc analyses: * indicates P<0.05.

The second morphological index described variation in body depth and the robustness of the caudal peduncle (Fig. 4). Negative scores described fish that were ventrally convex, and deeper bodied in the midsection, at the approximate location of the pelvic spine. Fish with negative scores had shorter but broader caudal peduncles. The relative positions of the pelvic and dorsal spines differed between fish with negative and positive scores; negative scores described fish with pelvic spines located to the posterior of the first dorsal spine while in fish with positive scores the pelvic and first dorsal spine were more or less vertically aligned. Again, morphology varied between sites (one-way ANOVA: F_(5, 215)_ = 133.99, P<0.001).

Negative scores were typical of fish from the estuary, which differed significantly from fish from all of the other sites (Tukey post-hoc, P<0.001). Fish from the two river sites had positive scores, describing shallower bodies and longer, narrower caudal peduncles. Fish from these two sites did not differ significantly from each other (P = 0.59), but differed significantly from fish from all other sites (P<0.001). Fish from the salt marsh and ditch sites scored intermediately and did not differ from each other significantly (salt marsh vs. ditch sites 1 and 2 respectively: P = 0.34 and 0.14; ditch site 1 versus ditch site 2, P = 0.98).

#### Morphological index 3

The third morphological index also described variation in the form of the caudal peduncle, as well as the degree of tapering posterior of the pelvic spine. Negative scores described caudal peduncles that were relatively short, while those in fish with positive scores were more elongated with a slight downward curve. In fish with negative scores we also saw elongation of the posterior section of the body, in the region of the anal and dorsal fins relative to those with positive scores. We also saw variation in the size and angle of the base of the pectoral fin; fish with negative scores possessed pectoral fins with broader bases that were angled vertically; in fish with positive scores the base of the pectoral fin was larger and angled forward.

**Figure 5:**
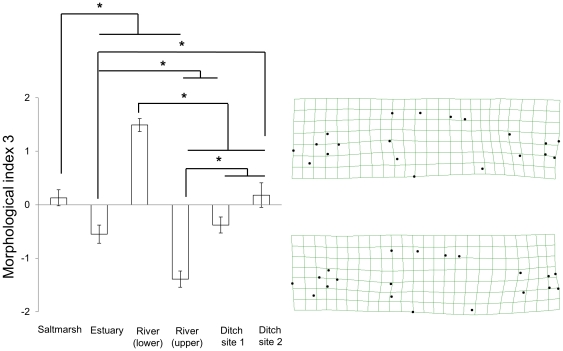
Morphological index 3. The mean scores (+/− standard error) of fish from each of the six collection sites for the third morphological index, describing described 22.1% of the morphological variation in the sample. Morphological index 3 describes variation in the form of the caudal peduncle, as well as the degree of tapering posterior of the pelvic spine. The deformation plots on the right of the figure show the morphologies associated with the positive-most and negative-most scores. Tukey post-hoc analyses: * indicates P<0.05.

Once again, morphology varied significantly between sites (one-way ANOVA: F_(5, 215)_ = 35.35, P<0.001, Fig. 5). Negative scores were most apparent in fish from the upper river site, and to a lesser extent in those from the estuary and first ditch site. Fish from the upper river site differed significantly from those from the other sites (Tukey post-hoc: P = 0.028 versus the estuary site, P<0.001 all other comparisons). Fish from the salt marsh and the two ditch sites did not differ significantly (P = 0.19; 0.99; 0.20, salt marsh versus ditch sites one and two and ditch site one versus ditch site two respectively). Fish from the lower river site had strongly positive scores and differed significantly from fish from all the other sites (P<0.001 in all comparisons).

### Dorsal and pelvic spine relative size

The sizes of the first dorsal and left pelvic spine were seen to be only weakly positively correlated (Pearson correlation, pooled across sites: n = 216, r = 0.30). Dorsal and pelvic spine sizes were therefore analysed separately.

**Figure 6:**
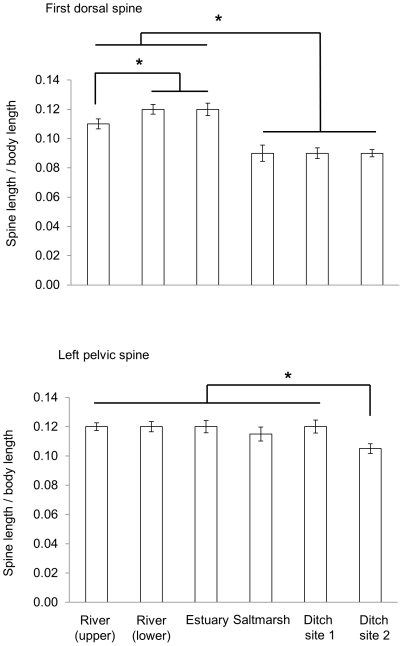
Dorsal and pelvic spine relative size. The relative spines lengths (spine length/body length; mean +/− standard error) of fish from each of the six collection sites. Tukey post-hoc analyses: * indicates P<0.05.

First dorsal spine length varied significantly between sites of collection (F_(5, 207)_ = 7.69, P<0.001), but did not co-vary with any of the first three morphological index scores (F_(1, 207)_ = 0.65, P = 0.42; F_(1, 207)_ = 0.17, P = 0.68 and F_(1, 207)_ = 0.88, P = 0.35 respectively). Fish from the estuary and lower river had proportionally longer spines than did those from the upper river, while fish from all three of these sites had longer spines than those from the two ditch sites and the salt marsh (Tukey HSD: P<0.001, Figure 6).

Pelvic spine length also varied significantly between sites (F_(5, 207)_ = 9.47, P<0.001), but again did not co-vary with any of the first three morphological indices (F_(1, 207)_ = 1.27, P = 0.21; F_(1, 207)_ = 0.03, P = 0.86; and F_(1, 207)_ = 0.04, P = 0.84 respectively). Fish from the salt marsh had proportionally shorter pelvic spines than fish from the other sites (Tukey HSD: P<0.001, Figure 6).

## Discussion

### General discussion

There was significant variation in the morphology of threespine sticklebacks collected from connected habitats within a single drainage basin that were separated by distances of at most <20 km and in some cases just hundreds of meters. In line with our predictions, sticklebacks from habitats where fish predators were absent had proportionally smaller first dorsal spines (ditch and salt marsh habitats) and pelvic spines (the salt marsh fish only). However, while there was also variation in body depth, as predicted, it was not clearly related to the presence of predatory fishes, since fish from the estuary and the upper river, both high predation sites (with respect to fish predators), had respectively the deepest and shallowest bodies across the sample. Body depth may depend upon complex interactions between predation pressure, flow rate and habitat structural complexity [44]. Similarly, we saw variation in caudal peduncle structure that was not apparently linked to channel flow rate. Finally, there was striking variation in orbit diameter. We discuss this variation in more detail below.

Morphological variation both in other populations of sticklebacks as well as in other fish species can be brought about by genetic factors, through phenotypic plasticity and through a combination of both [20], [25], [45], [46]. While we have no data on the underlying causes of the morphological variation seen in our population, it seems less likely that the observed variation is driven by significant genetic differences between fish from the different habitats. The four habitats we considered in our study are in close proximity to each other, with no physical barriers to gene flow. Fish are likely to move between habitats, both in order to breed [47], and because some habitats (the ditches and salt marshes) occasionally dry out completely and are presumably repopulated following re-flooding and reconnection to the river and estuary. It is possible, however, that there are other barriers to gene flow, for example pre- and post-zygotic mate choice [47], while local differences in water chemistry or other environmental conditions might constitute barriers to fish from different habitats [33]. We speculate that phenotypic plasticity may be more important in determining the variation in body shape seen here. Studies of the effects of rearing environment and resource use during ontogeny have found that phenotypic plasticity can account for a sizeable proportion of morphological variation in fishes of a number of species [20], [25], [45], [46]. Further work is needed in order to identify both the underlying causal mechanisms of the between-habitat morphological variation described in our study and the extent to which they represent ecological specialization, if at all [48]. Despite this, it is possible to speculate on these in light of the large literature on morphological variation and divergence in threespine sticklebacks and other fish species. Subjects of variation in our study included orbit diameter, body depth and the relative size of the dorsal and pelvic spines, and we discuss these below. Finally, habitat specific variation in morphology also has implications for our understanding of the behavioral ecology of generalist species living across multiple habitat types, and we briefly discuss this with regard to social organization, site fidelity and homing behavior.

### Accounting for the observed morphological variation

#### Orbit diameter

The first morphological index revealed by the DFA in our study, and accounting for around 37% of the variation seen in our sample included significant variation in orbit diameter between fish from different habitats. Proportionally larger eyes are seen in the plankton feeding limnetic specialist sticklebacks occurring in several lakes in British Columbia and Alaska [13], [14]. No data exists on the feeding ecology of sticklebacks in the Great Eau drainage, though this could be determined, for example, using stable isotope analyses [35], [49]. Larger eyes were also seen in populations of the phenotypically plastic Galaxid fish *Aplochiton zebra* from highly turbid lakes [17]. A previous study [42] focusing on the foraging efficiency of sticklebacks from the Great Eau drainage revealed that the smaller-eyed ditch fish forage with intermediate efficiency (relative to fish from the estuary, river and salt marsh habitats) under conditions of increasing turbidity. It was also found that chemical cues were at least as important as visual cues when foraging for sticklebacks from all four of the studied habitats, a conclusion that does not support the idea that variation in orbit diameter necessarily reflects adaptation to foraging in turbid environments. Further research is clearly required in order to determine the significance of the observed habitat specific differences in orbit diameter.

#### Body depth

The second and third morphological indices obtained from the DFA included significant between-habitat variation in body depth, ranging from relatively shallow-bodied fish from the two river sites, to the estuary, where the fish had the greatest body depth. Fish collected from the ditches and salt marsh were of intermediate body depth. In many aquatic vertebrates increased body depth has been demonstrated to be an adaptive response to predation from gape-limited predators [15], [16], [36], [37], [38], [50]. Gape-limited predators ingest their prey intact, and the maximum size of potential prey is therefore determined by the minimum dimension of the predator's mouth and oesophageal tract.

Gape-limited predators have been shown to take longer to handle and swallow deeper bodied prey compared to shallower bodied prey, affording a greater window of opportunity for escape, and some predators therefore preferentially select shallow-bodied over deeper-bodied prey when both are available [51]. As such there is a strong selection pressure favoring deeper bodied phenotypes in prey populations that are subject to predation from gape-limited predators. A significant cost of developing a deep body is an increase in hydrodynamic drag and the associated energetic losses [52], [53]. Since predation pressure and the energetic costs of locomotion (which might depend, for example, upon flow rate) can vary across habitats, the balancing point of this trade-off is dynamic. As a consequence, many aquatic prey species of gape limited predators possess a capacity for plasticity in body depth, with the development of deeper bodies only being induced by exposure to predator specific chemical stimuli. Such plasticity has been documented in both fishes (although not in sticklebacks) [15], [16], [50] and larval anurans [37], [38]. It is feasible that a similar mechanism operates in sticklebacks, however to our knowledge this remains to be explored.

In our study, body depth was shallower in the fish collected from the two river channel sites, where flow rate was greater than in the estuary or ditches. Furthermore the deeper bodied estuary fish also had more robust caudal peduncles. Although sticklebacks are labriform swimmers, propelled primarily by pectoral sculling, they also use their caudal fins for propulsion, especially in fast-start swimming [54].The observed morphological characteristics may suggest adaptations to increase swimming power in response to the increased hydrodynamic drag brought about by greater body depth. While this finding is broadly consistent with a body depth trade-off between flow rate and predation risk, the link remains to be demonstrated categorically. Although predation pressure is likely to be high in the estuary of our study drainage, a similar assemblage of fish predators also occurs in the non-tidal reaches of the river, and it is unclear to what extent predation intensity varies between habitats. Habitat complexity, such as substrate structure and vegetation density also varies between these locations, and undoubtedly also has an effect upon flow rate.

Body depth may also be linked to feeding mode. Spoljaric & Reimchen [23] report that more streamlined bodies are typical of sticklebacks found in deep lakes and feeding predominantly on zooplankton, while those found in small shallow lakes and feeding on benthic prey possess deeper bodies. They suggest that the shallow body of planktivores may be an adaptation for the high mobility required to maximize encounter rates with patchily distributed prey in open water. At present we have no data on prey use in our focal population. Further research is needed if we are to gain a clearer understanding of the likely complex relationship between swimming energetics, habitat structure, feeding mode, predation regime, and body depth variation in this species.

#### Dorsal and pelvic spines

Like environmentally induced plasticity in body depth, stickleback spines are an evolved countermeasure to predation from gape limited fish and bird predators. By erecting its spines the stickleback can increase its effective body diameter, preventing itself from being swallowed, or else increasing the handling time of the predator, prolonging its window of opportunity for escape [55]. Spines are costly to produce however, since their development requires the sequestration of resources that might otherwise be invested in growth or reproduction. Defensive spines and similar structures are a common response to predation from gape-limited predators and plasticity in the presence, size and number of such structures in response to predation pressure has been described in species from a range of taxa [5], [39], [40], [56], [57]. In sticklebacks, populations under low or no predation pressure exhibit reduced spine size, and in some cases a complete absence of spines and bodily armor [39], [40]. Consistent with this, we saw that the length of the first dorsal spine was proportionally lower in fish from the ditch and salt marsh sites, which contain no fish predators, compared to the river and estuary sites, while pelvic spines were also proportionally shorter in the salt marsh fish.

While it was possible to document the presence or absence of fish predators, it is likely that predation from birds (including grey heron, *Ardea cinerea*; kingfisher, *Alcedo atthis* and little egret, *Egretta garzetta*) occurs at all sites to some extent. As endotherms, predatory birds almost certainly consume more prey per unit of body mass than do predatory fish and this likely increases during colder months. Predation from birds may therefore be more important than predation from fishes in some or all locations at our study site, while the impact of avian predation may interact with local variation in depth, turbidity, vegetation coverage and season.

#### Sexual dimorphism

Recently, a number of studies have described sexual dimorphism in threespine sticklebacks [58], [59], [60], [61], [62]. Typically this is seen in head size, with males having a larger, proportionally longer heads than females [58], [59], [60], [62], while females can have longer pelvic girdles [60]. Aguirre & Akinpelu [59] reported this pattern in each of ten populations originating from anadromous, stream and lake environments, while Spoljaric & Reimchen [62] described males with larger heads, a larger gape, longer anal fin, and deeper posterior caudal depth compared to females in fish from 52 lake, stream and marine sites. Kitano et al. [60] established that sexual dimorphism with respect to head size has a genetic basis. Reimchen & Nosil [61] report that for one population males and females differ in the extent to which they use open water niches, ecological variation which may explain some of the observed morphological variation between the sexes.

In our study, the sex of the fish was not known. It is unfortunate that this potential source of variation cannot be accounted for, however we have no reason to believe that sexual dimorphism might underlie the habitat-specific morphological variation described in our study. While we saw variation in orbit diameter, we did not see the kind of intersexual variation in relative head size or elongation described in the above cited studies. We can think of no plausible mechanism for habitat-biased sex distributions, such as male nest site location preferences, or sex-biased post-mating mortality, since the fish used in our study we collected in late summer, after the breeding season had finished and would have been unmated young of the year, spawned several months previously in the spring. Nevertheless, future studies at this site will include subject sex as a variable.

### Implications for behavioral ecology

The high-levels of habitat-specific morphological variation seen in our study imply either assortive mating and limited gene flow between adjacent habitats, or that the variation is driven primarily by phenotypic plasticity. In either case, local functional trade-offs with respect to morphology may restrict the freedom of individuals to move between populations, since any immigrants would likely be at a competitive disadvantage relative to residents. In other fish species, where morphology is known to correspond to resource specialization, such as in some pelagic and littoral polymorphic species, individuals suffer significant fitness costs associated with lower foraging efficiency and competitive disadvantage when moving out of the habitat to which they are adapted and entering other areas [21], [22], [24]. It would be useful to determine whether similar fitness trade-offs are also exist with respect to movement between habitats in our study system.

If such fitness costs exist among the sticklebacks in our study system they should promote site or habitat fidelity. One mechanism by which this could be achieved is through self-referent matching of resource derived cues in shoaling decisions. Recent work has shown that a number of shoaling fish species, including sticklebacks, preferentially shoal with others that have recently eaten the same foods or been exposed to the same environmental conditions as themselves [63], [64], [65], [66], [67], [68], [69], [70]. Further, this mechanism of social organization has been shown to operate among sticklebacks in the field in our study drainage with individuals showing shoaling preferences for conspecifics from their own location over those from other areas [66]. These preferences were seen both within and between habitats, with fish being able to discriminate between those from their own point of capture and from locations less than half a kilometer away. Conceivably, one factor driving the evolution of self-referent shoaling behavior could be adaptation to local conditions, and the subsequent fitness costs of moving into the ‘wrong’ habitat and this idea merits further research. Social organization and the distribution of individuals through the environment, by whatever mechanisms it operates, influences social and competitive interactions, determines the rate and patterns of transmission of information and pathogens and can affect patterns of mating, all of which have implications for the ecology and evolution of species.

### Conclusions

Body shape varied significantly among threespine sticklebacks from separate but interconnected habitats within a single drainage basin. Variation included differences in orbit diameter, body depth, caudal peduncle shape, pectoral fin positioning and relative dorsal and pelvic spine size. Interestingly, one of the habitats, the ditch system, is entirely artificial and is managed through regular dredging, suggesting that human management of habitats can in some circumstances lead to morphological variation among the animals that inhabit them. Though an ecologically generalist species, the threespine sticklebacks in our study system were seen to exhibit lower variation in body shape within habitats than between them. Whether this indicates local, inter-habitat functional trade-offs remains to be determined. Finally, while we speculate that phenotypic plasticity rather than genetic differentiation is likely to be the major driving force behind the observed variation, further work is required in order to determine their relative importance.
